# Bidirectional mendelian randomization identifies plasma proteins associated with cervical cancer risk

**DOI:** 10.1186/s43046-026-00374-7

**Published:** 2026-07-01

**Authors:** Yan-Hong Zhao, Qing-Fen Ruan, Jing-Hua Ning, Xin Zhang, Run Qu, Jing Zou, Yi Liang, Cheng-gui Zhang, Yu-Zhe Zhang

**Affiliations:** 1 Dali University, Dali, China; 2 The First Affiliated Hospital of Dali University, Dali, China; 3 University Health Network, Toronto, Canada

**Keywords:** Plasma proteins, Cervical cancer, Mendelian randomization, Tumor markers

## Abstract

**Background:**

Cervical cancer continues to pose a considerable challenge to global health, necessitating innovative approaches for improved diagnostics and personalized treatment strategies. Prior investigations have suggested that plasma proteins may play a role in the pathogenesis of cervical cancer; however, these studies do not confirm a causal relationship. To address this gap, conducted a large-scale Mendelian randomization (MR) study of the plasma proteome.

**Methods:**

We performed a two-sample bidirectional Mendelian randomization analysis involving 4,907 plasma proteins, utilizing publicly accessible genome-wide association study (GWAS) summary statistics, to examine the causal association between the plasma proteome and the risk of cervical cancer. Analytical methods included inverse variance weighting (IVW), weighted median, MR-Egger regression, and simple and weighted models. Additionally, we performed sensitivity analyses to evaluate heterogeneity and horizontal pleiotropy through Cochran's Q test, MR-Egger intercept, MR-PRESSO test, and leave-one-out analysis. We also applied false discovery rate (FDR) correction to the results of all IVW methods to identify the plasma proteins most strongly associated with cervical cancer. Finally, we enriched the most relevant plasma protein genes using the Kyoto Encyclopedia of Genes and Genomes (KEGG) and Gene Ontology (GO) analyses and GeneMANIA to identify disease-related pathways.

**Results:**

According to the IVW method, seven plasma proteins are significantly associated with cervical cancer risk (*P* < 0.05). Specifically, six proteins demonstrated protective factors: DEFB135 (OR = 0.201, 95% CI = 0.082–0.492, *P* < 0.001), FGL2 (OR = 0.104, 95% CI = 0.032–0.338, *P* < 0.001), FTMT (OR = 0.612, 95% CI = 0.465–0.804, *P* < 0.001), PDIA4 (OR = 0.088, 95% CI = 0.026–0.295, *P* < 0.001), SPHK2 (OR = 0.102, 95% CI = 0.030–0.350, *P* < 0.001), and TMED2 (OR = 0.045, 95% CI = 0.008–0.246, *P* < 0.001). In contrast, RACGAP1 (OR = 1.755, 95% CI = 1.286–2.395, *P* < 0.001) was identified as a risk factor. Reverse MR analysis revealed no significant evidence of reverse causation (*P* > 0.05) between cervical cancer and these plasma proteins. Functional enrichment analysis identified several biologically relevant pathways potentially involved in cervical cancer pathogenesis, including the establishment of organelle localization, regulation of oxidoreductase activity, Ferroptosis, and Porphyrin metabolism.

**Conclusion:**

These findings suggest that DEFB135, FGL2, FTMT, PDIA4, SPHK2, and TMED2 may protect against cervical cancer, while RACGAP1 may represent a potential risk factor. The identified tumor markers provide mechanistic insights into the molecular basis of cervical cancer and warrant further investigation in functional studies.

**Supplementary Information:**

The online version contains supplementary material available at 10.1186/s43046-026-00374-7.

## Introduction

Cervical cancer (CC) is the leading cause of cancer-related deaths among women worldwide. Recent epidemiological data from the International Agency for Research on Cancer (IARC) estimate that its incidence and mortality rates are particularly significant among common female cancers [1]. The primary risk factor for cervical cancer is persistent infection with high-risk subtypes of human papillomavirus (HPV). The E6 and E7 proteins of HPV promote the malignant transformation of cells and tumorigenesis by interacting with host cell proteins [2]. Current therapeutic strategies for cervical cancer rely heavily on platinum-based chemotherapeutic agents, such as cisplatin; however, the emergence of drug resistance frequently compromises treatment efficacy and contributes to disease recurrence [3]. The World Health Organization (WHO) emphasizes that early detection and timely intervention are critical for reducing the burden of cervical cancer [4]. Therefore, Screening for potential targets to combat cervical cancer is essential.

In recent years, plasma proteins have emerged as significant targets for drug development due to their ability to diagnose and predict diseases, identify therapeutic targets, and elucidate the pathophysiology of various conditions [5]. Previous studies have demonstrated the high independent diagnostic value of squamous cell carcinoma antigen (SCCA) as a tumor biomarker for cervical cancer [6]. CA125 has been recognized as a biomarker for the diagnosis and prognosis of cervical cancer [7]. Furthermore, A study based on the UK Biobank cohort found that the PAX8, CLPTM1L, and HLA genes play a role in cervical carcinogenesis [8]. However, traditional observational studies are often limited by confounding factors and reverse causality, leading to inconclusive findings. To address these limitations. Recent research has integrated protein quantitative trait loci (pQTL) into Mendelian randomization (MR) analyses, enabling the prioritization of drug targets with enhanced causal inference [9]. Mendelian randomization utilizes single-nucleotide polymorphisms (SNPs) as instrumental variables to infer causal relationships between exposure factors and outcomes [10], minimizing bias from confounding factors and avoiding interference from reverse causation [11].

With the rapid advancement of high-throughput proteomics techniques in plasma analysis, researchers have established a causal relationship between plasma proteins and various diseases, including multiple sclerosis, acne, and breast cancer, through MR analysis [12–14]. However, the causal relationship between plasma proteins and cervical cancer remains poorly understood. Therefore, we leveraged the largest available plasma proteomics dataset to perform a comprehensive two-sample bidirectional MR analysis, examining the causal effects of 4,907 plasma proteins on cervical cancer risk. Our findings provide novel insights into the molecular mechanisms underlying cervical cancer and establish a theoretical base for advancing early screening, diagnostic strategies, and therapeutic interventions for this disease.

## Materials and methods

### Study design

**Fig. 1:**
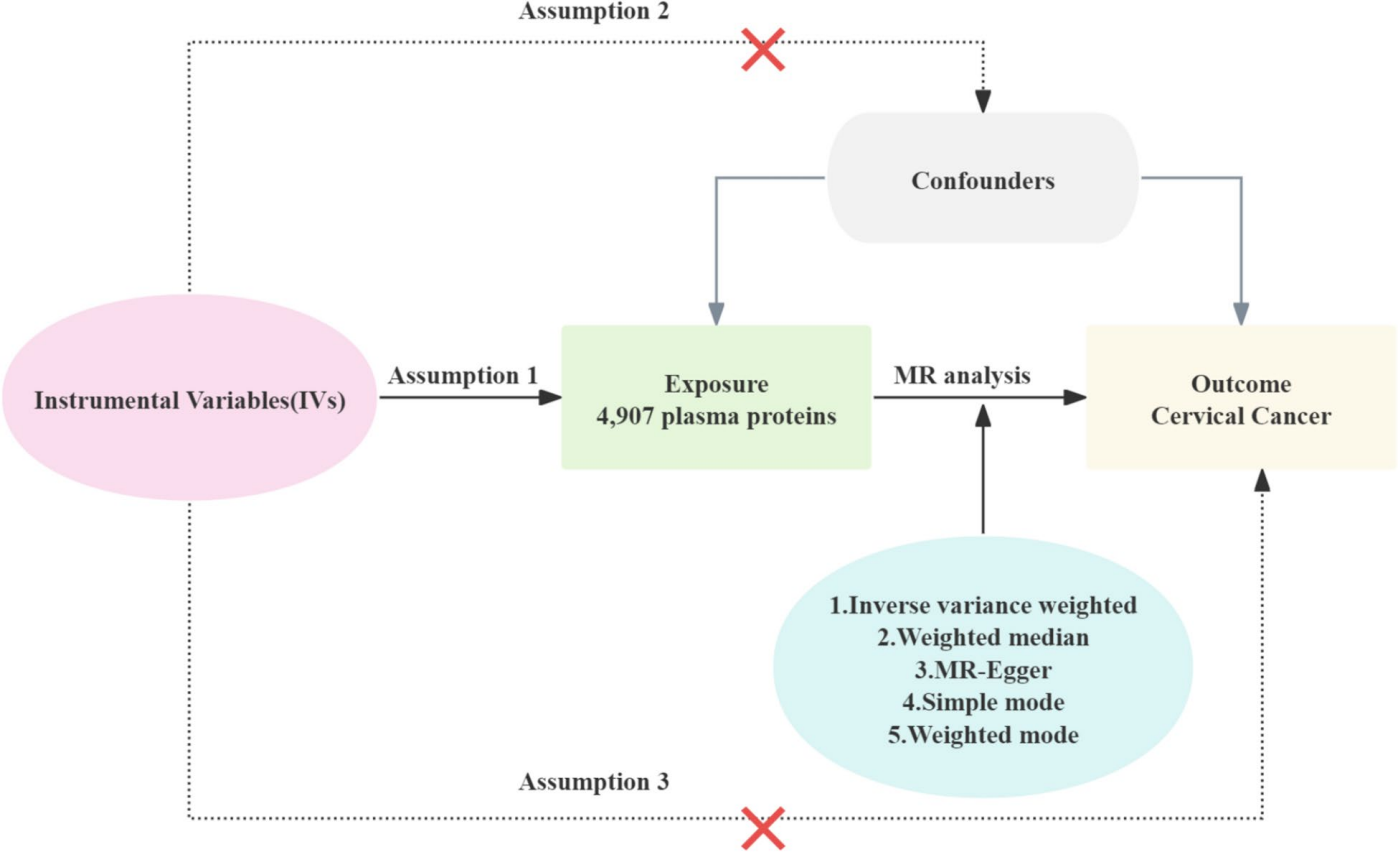
Fundamental assumptions of Mendelian randomization (MR) analysis. Relevance assumption (1) Instrumental variables (IVs) must strongly associate with the exposure. Independence assumption (2): IVs must be independent of confounding factors influencing the exposure and the outcome. Exclusion restriction assumption (3): IVs must affect the outcome exclusively through exposure, with no alternative pathways

**Fig. 2:**
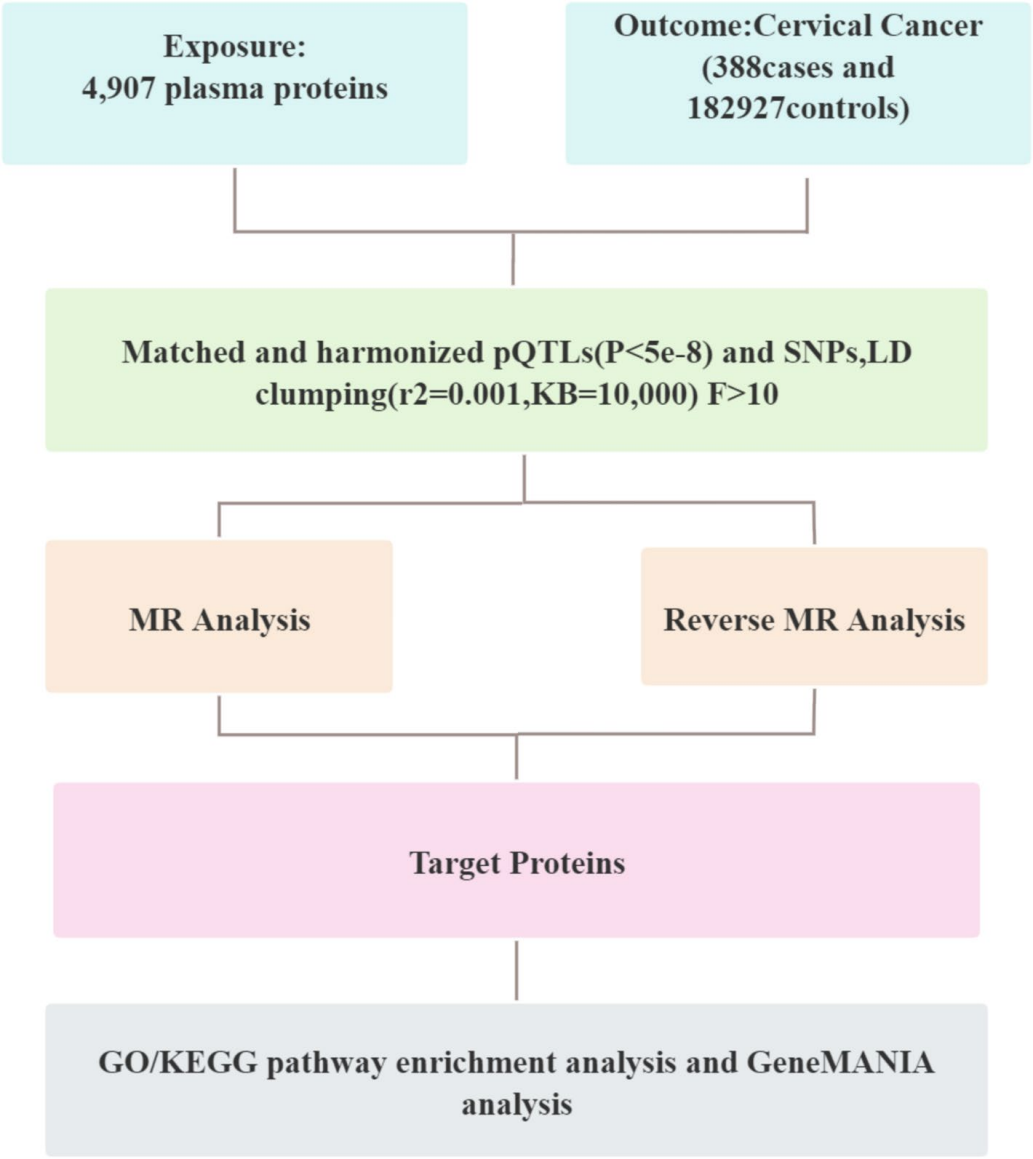
Flowchart of the study design

We employed a two-sample bidirectional MR method to investigate the causal relationship between 4,907 plasma proteins and cervical cancer. Furthermore, we conducted extensive sensitivity analyses to ensure our findings were robust and reliable. Finally, functional enrichment and GeneMANIA analyses were performed on the plasma protein-coding genes most significantly associated with cervical cancer. Large-scale proteomics data were analyzed using R software (v 4.3.2).MR analysis relies on three fundamental assumptions: (a) the relevance assumption, instrumental variables (IVs) must be closely related to the exposure factors; (b) the independence assumption, instrumental variables should be independent of any confounders related to both the exposure and the outcome; (c) the exclusion restriction assumption: instrumental variables should only influence the outcome through the exposure factors, rather than acting directly on the outcome (Fig. 1). The study design is presented in (Fig. 2).

### Data sources

The exposure data utilized in this study were obtained from a publicly accessible proteomics genome-wide association study (GWAS) dataset available through the deCODE database (https://www.decode.com). This dataset is derived from a comprehensive pQTL analysis conducted on a cohort of 35,559 Icelandic individuals, from which 4,907 plasma proteins were identified (Supplemental Table 1) [15].

**Table 1:** The data sources utilized for the Mendelian randomization analysis within the study

	Database	Sample size	Population
Plasma protein	deCODE	4907	Icelander
Cervical cancer	FinnGen	388	European

GWAS data for cervical cancer (GWAS ID: finngen_R10_C3_CERVIX_UTERI_EXALLC) were obtained from the Finnish database (https://www.finngen.fi/en/access_results). Specifically, we utilized cohort data labelled as "Malignant neoplasm of the cervix (controls excluding all cancers)," which included 388 cases and 182,927 controls (R10) (Table 1) (Supplemental Table 2). To minimize bias due to population heterogeneity, we restricted the analysis to individuals of European descent [16]. Additionally, the relevant ethical committees approved all data used in this study, and no further ethical.

**Table 2:** Sensitivity analysis of the forward Mendelian randomization (MR) study with cervical cancer (CC) as the outcome

	**Heterogeneity analysis**						**Pleiotropy analysis**		
	**Inverse variance weighted**			**MR Egger**			**MR Egger**		
	**Q**	**Q_df**	**Q_pval**	**Q**	**Q_df**	**Q_pval**	**Egger_intercept**	**se**	**pval**
DEFB135	2.7523	4	0.6000	1.8838	3	0.5969	0.1151	0.1151	0.4201
FGL2	3.5316	3	0.3167	2.9013	2	0.2344	0.1726	0.2618	0.5776
FTMT	5.5965	10	0.8479	5.5133	9	0.7875	−0.0166	0.0574	0.7795
PDIA4	2.9094	3	0.4058	2.5884	2	0.2741	0.1525	0.3062	0.6679
RACGAP1	9.5805	11	0.5685	9.0124	10	0.5309	0.0463	0.0614	0.4684
SPHK2	2.0807	3	0.5558	1.4094	2	0.4942	0.1922	0.2346	0.4987
TMED2	2.4461	2	0.2943	2.4457	1	0.1178	−0.0052	0.4426	0.9926

approvals were necessary.

### Selection of instrumental variables

To identify valid IVs, we implemented rigorous selection criteria using the TwoSampleMR package in R (v 4.3.2). First, to satisfy the relevance assumption of MR analysis, we selected SNPs that were closely associated with the exposure, applying a threshold of *P* < 5 × 10^^−8^ [17]. Second, to guarantee the independence of each IV, linkage disequilibrium (LD) is minimized. This is accomplished by pruning SNPs using a threshold of r^2^ < 0.001 within a genomic window of 10,000 KB. Third, an F-statistic calculated as F = (R^2^·n)/(1-R^2^) exceeding a threshold of 10 is employed to exclude weak instrumental variables, thereby reducing the bias attributable to weak instruments. Finally, we excluded SNPs with palindromic structures when harmonizing exposure and outcome data using the harmonise_data function [18]. For reverse MR analysis, we applied a significance threshold of *P* < 5 × 10^^−6^ to identify SNPs strongly associated with cervical cancer (CC). In contrast, for forward MR analysis, a threshold of *P* < 5 × 10^^−8^ was sufficient to ensure adequate plasma protein IVs.

### Forward Mendelian randomization analysis

To assess the causal relationship between 4,907 plasma proteins and cervical cancer, we applied five distinct MR methods. The inverse variance weighted (IVW) method integrates the Wald ratio estimates of the causal effects of various SNPs to assess the causal relationship between exposure and outcome, making it the predominant analytic method [19]. The weighted median is more robust to invalid instrumental variables, providing reliable estimates as long as valid instruments contribute more than 50% of the total weight [20]. The MR-Egger regression method evaluates invalid causal hypotheses and ensures consistency in assessing causality, particularly in cases where instrumental variables exhibit insufficient genetic variation [21]. The weighted mode effectively captures the most representative causal relationships by emphasizing the effect values of the plurality, especially when multiple instrumental variables are involved. Additionally, the simple mode is another analytical method based on the effects of plurality, which is more resilient to extreme values and less influenced by small amounts of outlier data. When different analytical methods yield inconsistent results, the IVW method should be prioritized.

### Sensitivity analysis

Sensitivity analyses were conducted to ensure the robustness and reliability of our findings. These included Cochran's Q test, MR-Egger intercept, MR-PRESSO, and leave-one-out analysis. The heterogeneity of the findings was analyzed using Cochran's Q test; a *P* > 0.05 indicates the absence of heterogeneity [22]. The pleiotropy of relationships between instrumental variables and other potential confounders was assessed through the MR-Egger method. A result of *P* < 0.05 in the MR-Egger intercept analysis suggests the presence of horizontal pleiotropy. Additionally, the MR-PRESSO method was explicitly employed to identify outlier SNPs that exhibited horizontal pleiotropy, provided that more than 50% of the instrumental variables were valid [23]. These comprehensive sensitivity analyses collectively strengthened the validity of our causal inferences.

Furthermore, we conducted a leave-one-out analysis, which reassessed the effect values of the remaining SNPs by sequentially removing each SNP. This approach aimed to validate the results' reliability and robustness by evaluating each SNP's impact on the findings. Scatter plots were generated to visually represent the causal relationships between genetic instruments and outcomes. Additionally, forest plots were employed to display effect sizes and their corresponding statistical significance.

### Reverse Mendelian randomization analysis

To further elucidate the causal relationship between exposure and outcome, we conducted a reverse MR analysis along with a sensitivity analysis, using CC as the exposure factor and plasma proteins associated with CC, identified in the forward MR analysis, as the outcome. This approach facilitated the identification of potential reverse causality, mitigated the effects of confounding factors, and ensured the robustness of our study. The analytical and statistical methods employed were as previously described [24].

### GO/KEGG pathway enrichment and GeneMANIA analysis

To elucidate the biological relevance of the identified plasma proteins in CC, we performed functional annotation and pathway enrichment analyses using the clusterProfiler package (v 4.10.1) in R (v 4.3.2). Gene Ontology (GO) analysis was conducted to categorize the biomarker genes into three functional domains: biological processes (BP), molecular functions (MF), and cellular components (CC). Concurrently, the Kyoto Encyclopedia of Genes and Genomes (KEGG) pathway enrichment analysis was employed to identify metabolic and signaling pathways associated with these genes. A significance threshold of *P* < 0.05 was applied for both GO and KEGG analyses. Furthermore, to explore potential protein–protein interactions, we constructed a protein–protein interaction (PPI) network using GeneMANIA (http://genemania.org/), providing insights into the functional relationships among the identified biomarkers.

## Results

### Forward MR analysis

First, we screened exposed SNPs using a significance threshold of *P* < 5 × 10^^−8^, identifying 14,475 SNPs linked to plasma proteins as IVs. We then performed a two-sample MR analysis for each of the 4,907 plasma proteins using five distinct analytical methods. The results were further refined using the IVW method, applying a significance threshold of *P* < 0.05 and a false discovery rate (FDR) < 0.2. Ultimately, we identified seven plasma protein phenotypes significantly associated with CC: DEFB135, FGL2, FTMT, PDIA4, SPHK2, TMED2, and RACGAP1.

**Fig. 3:**
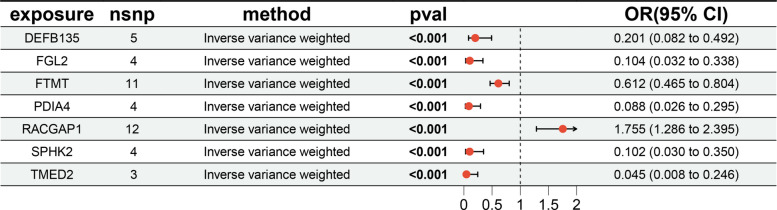
Forest plots illustrating the causal relationships between plasma protein phenotypes and cervical cancer (CC). Odds ratio (OR): An OR > 1 indicates a positive association between the plasma protein phenotype and CC risk, while an OR < 1 suggests a protective effect. Confidence interval (CI): The 95% CI reflects the precision of the effect estimate

**Fig. 4:**
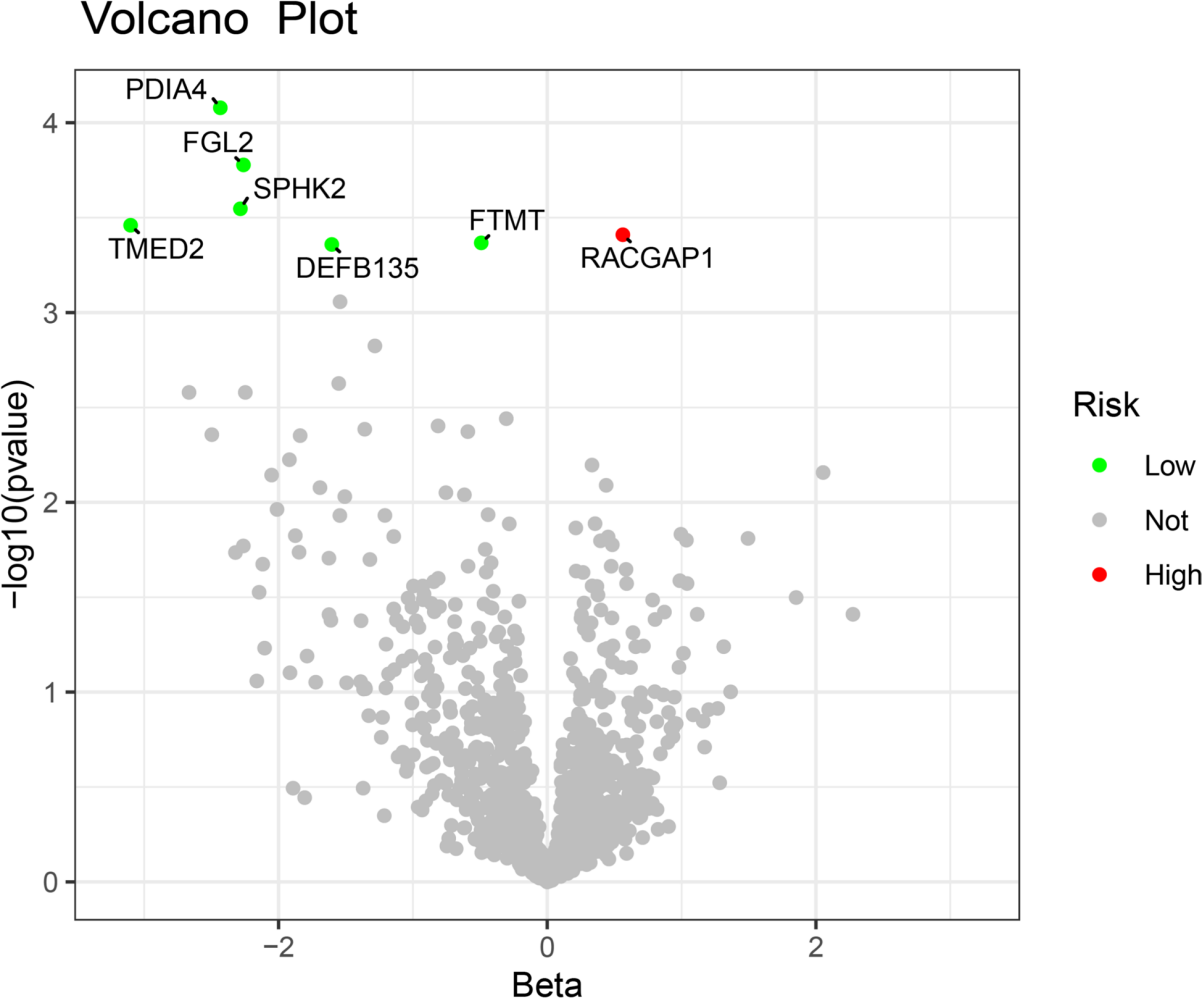
Volcano plot showing the results of the MR of the plasma proteins. Low: Plasma protein phenotype associated with a protective effect against cervical cancer (CC); Not: Plasma protein phenotype showing no significant causal impact on CC; High: Plasma protein phenotype associated with an increased risk of CC

The results of the IVW analysis indicated that DEFB135 (OR = 0.201, 95% CI = 0.082–0.492, *P* < 0.001), FGL2 (OR = 0.104, 95% CI = 0.032–0.338, *P* < 0.001), FTMT (OR = 0.612, 95% CI = 0.465–0.804, *P* < 0.001), PDIA4 (OR = 0.088, 95% CI = 0.026–0.295, *P* < 0.001), SPHK2 (OR = 0.102, 95% CI = 0.030–0.350, *P* < 0.001), and TMED2 (OR = 0.045, 95% CI = 0.008–0.246, *P* < 0.001) were negatively correlated with CC, suggesting a potential protective effect. Conversely, RACGAP1 (OR = 1.755, 95% CI = 1.286–2.395, *P* < 0.001) exhibited a positive correlation with CC, indicating its possible role in disease pathogenesis (Fig. 3). These findings were further supported by complementary analyses, including MR-Egger regression, weighted median (WM), and both simple and weighted mode methods. To enhance the robustness of our results, we generated volcano plots to visualize the strength and significance of the associations (Fig. 4).

### Sensitivity analysis

**Fig. 5:**
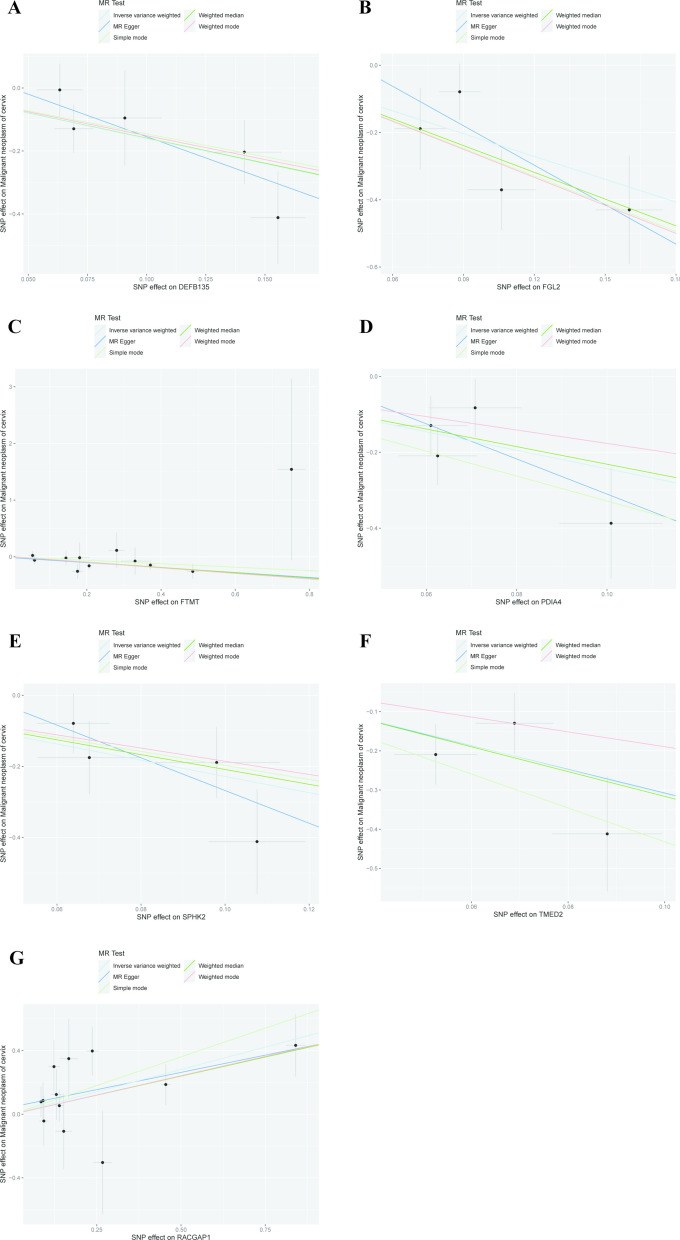
Scatter plots of Mendelian randomization (MR) analysis showing the association between seven plasma proteins (**a** DEFB135, **b** FGL2, **c** FTMT, **d** PDIA4, **e** SPHK2, **f** TMED2, and **g** RACGAP1) and cervical cancer (CC). X-axis: Genetic association with plasma protein levels. Y-axis: Genetic association with CC risk. The five lines represent the results from five distinct MR methods. Each black dot corresponds to a single nucleotide polymorphism (SNP) used as an instrumental variable

The robustness of our findings was validated through sensitivity analyses, including heterogeneity assessment using Cochran's Q test and evaluation of horizontal pleiotropy via the MR-Egger intercept test. The Cochran's Q test revealed that the p-values for the seven plasma proteins associated with CC were greater than 0.05, indicating no significant heterogeneity. Similarly, the *P*-values from the Egger-intercept test were also above 0.05, suggesting that they were not influenced by horizontal pleiotropy (Table 2). Further validation using the MR-PRESSO method confirmed the reliability of the MR results. Additionally, the leave-one-out method analysis demonstrated that our findings were not dependent on any single SNP (Fig. 5).

### Reverse Mendelian randomization analysis

**Fig. 6:**
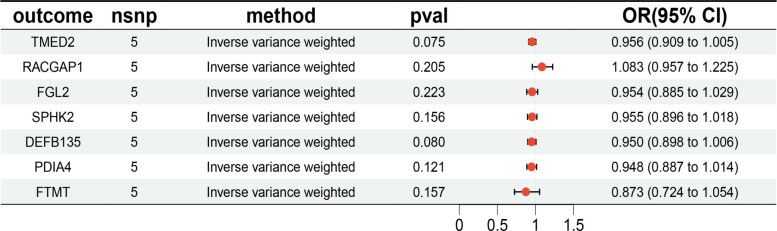
Forest plots illustrating the reverse causal relationships between plasma protein phenotypes and cervical cancer (CC). Odds ratio (OR): An OR > 1 indicates a positive association between the plasma protein phenotype and CC risk, while an OR < 1 suggests a protective effect. Confidence interval (CI): The 95% CI reflects the precision of the effect estimate

To assess potential reverse causality between the seven identified plasma proteins and CC, we performed an inverse MR analysis. This analysis included the IVW method, MR-Egger regression, weighted median, simple mode, and weighted mode. The IVW results indicated no significant causal effects of the plasma proteins on CC risk: TMED2 (OR = 0.956, 95% CI = 0.909–1.005, *P* = 0.075), FGL2 (OR = 0.954, 95% CI = 0.885–1.029, *P* = 0.223), SPHK2 (OR = 0.955, 95% CI = 0.896–1.018, *P* = 0.156), DEFB135 (OR = 0.950, 95% CI = 0.898–1.006, *P* = 0.080), PDIA4 (OR = 0.948, 95% CI = 0.887–1.014, *P* = 0.121), FTMT (OR = 0.873, 95% CI = 0.724–1.054, *P* = 0.157), and RACGAP1 (OR = 1.083, 95% CI = 0.957–1.225, *P* = 0.205). Similarly, none of the supplementary MR methods yielded statistically significant causal estimates (all *P* > 0.05) (Fig. 6), providing no evidence to support reverse causality between these plasma proteins and CC.

Sensitivity analysis of the reverse MR revealed Cochran's Q test *P*-values < 0.05, indicating the presence of heterogeneity, likely attributable to genetic variation influencing phenotypes and population-specific genetic backgrounds, potentially mediated through multiple pathways. Consequently, employing a random-effects IVW analysis can partially address the heterogeneity observed in the results. Moreover, the *P*-values for the Egger intercept and the MR-PRESSO global test were greater than 0.05, indicating no evidence of horizontal pleiotropy.

### GO/KEGG pathway enrichment and GeneMANIA analysis

In the GO analysis, the disease-associated plasma proteins were significantly enriched in specific BP, CC, and MF. For BP, the proteins were primarily involved in the establishment of organelle localization and the regulation of oxidoreductase activity. For CC, enrichment was observed in pathways such as the zymogen granule membrane, COPI-coated vesicle membrane, zymogen granule, COPI-coated vesicle, Flemming body, spindle midzone, cleavage furrow, Golgi-associated vesicle membrane, ER to Golgi transport vesicle membrane, and cell division site. For MF, the proteins were enriched in functions including ferric iron binding, oxidoreductase activity, acting on metal ions, bioactive lipid receptor activity, protein disulfide isomerase activity, intramolecular oxidoreductase activity, transposing S–S bonds, ferrous iron binding, protein-disulfide reductase activity, gamma-tubulin binding, frizzled binding, and phosphatidylinositol-3,4,5-trisphosphate binding. The number of plasma proteins enriched in each pathway exceeded one, as indicated on the abscissa, and all enrichments were statistically significant (*P* < 0.05). The highest confidence level was observed for biological processes (*P* < 0.01).

**Fig. 7:**
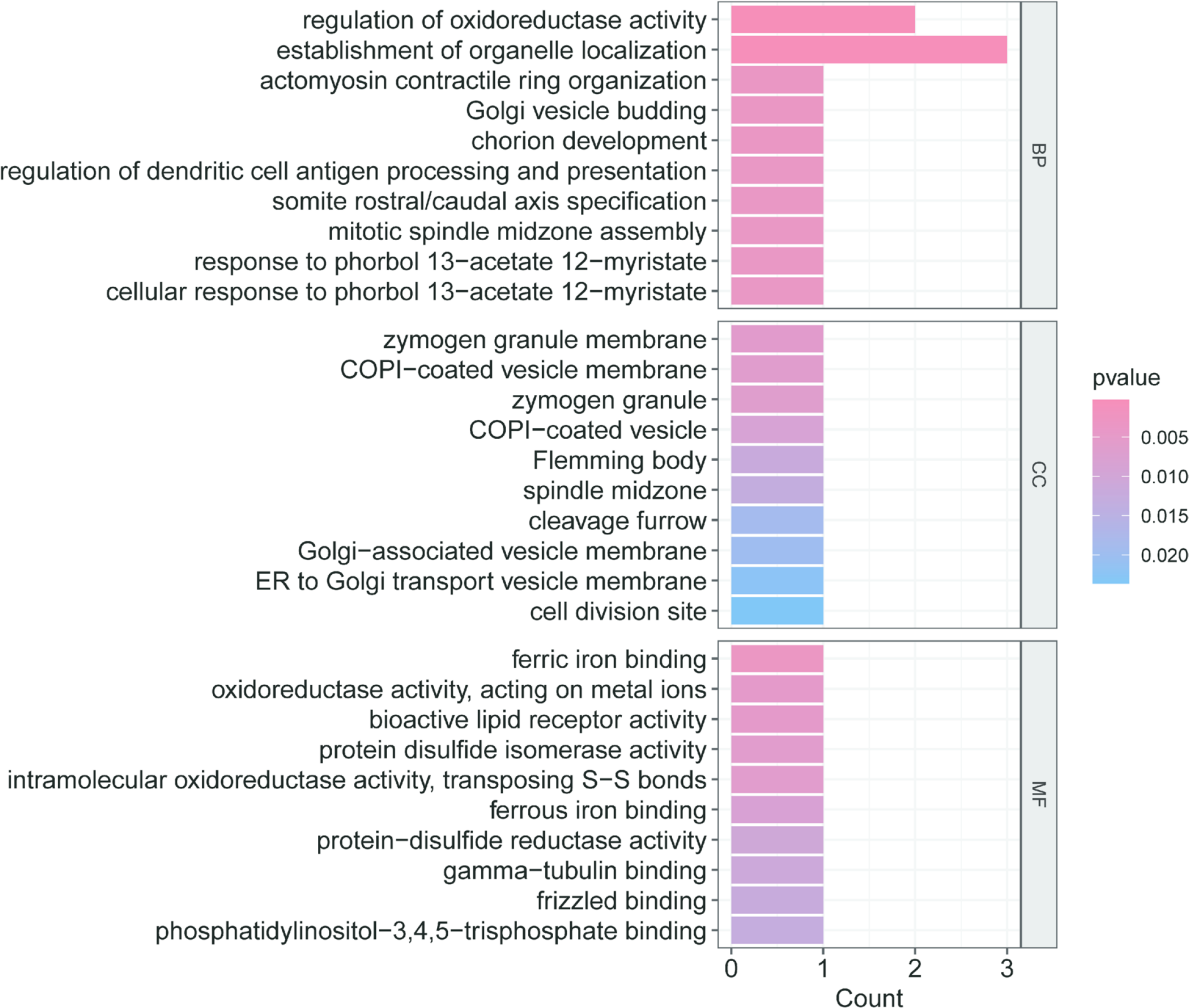
Bar chart of GO enrichment across biological processes (BP), cellular components (CC), and molecular functions (MF). The bar color represents the p-value, and "Count" indicates the number of enriched genes

**Fig. 8:**
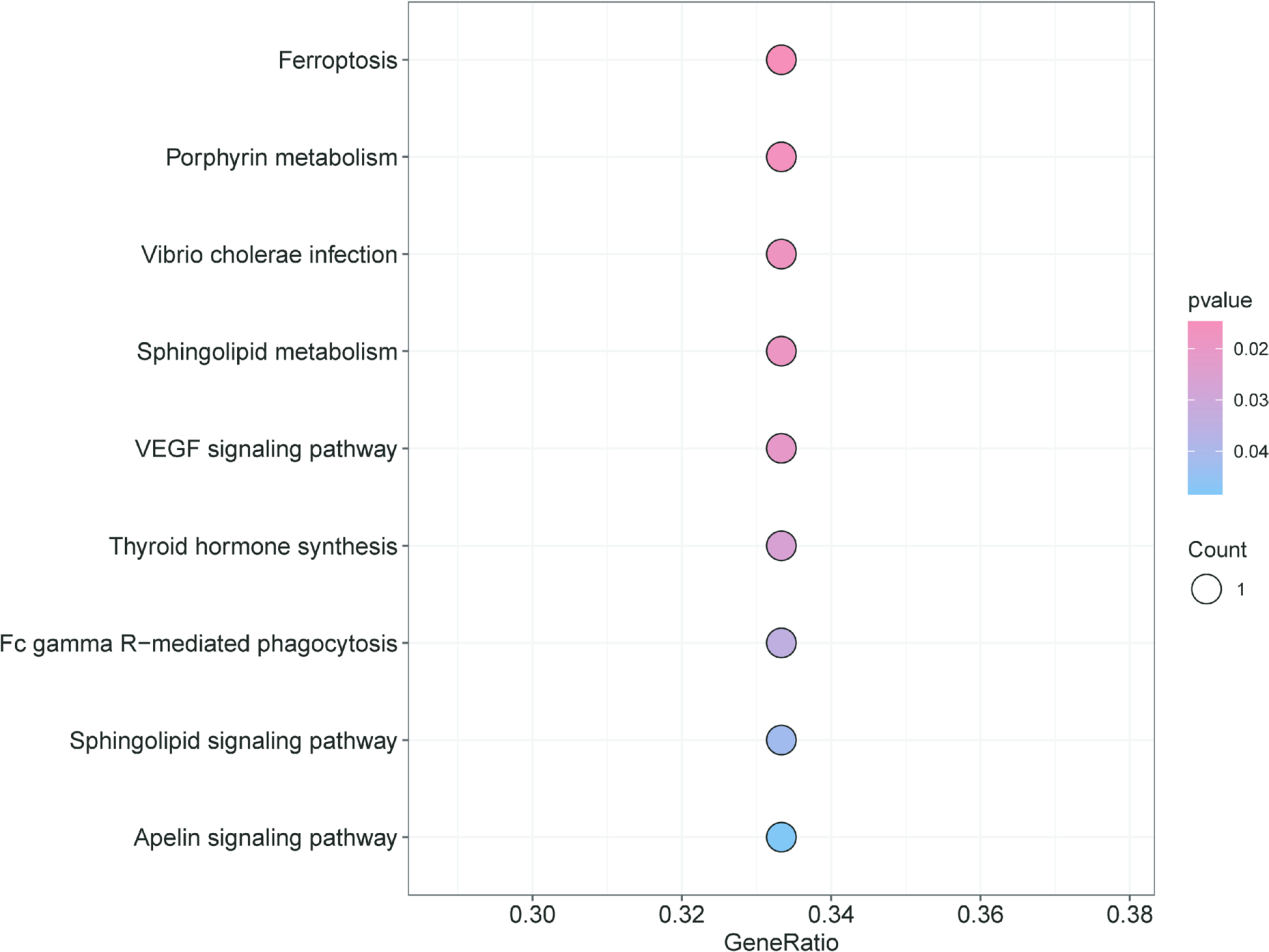
Bubble chart of KEGG enrichment. The bubble color corresponds to the *P*-value, and the bubble size reflects the number of enriched genes

**Fig. 9:**
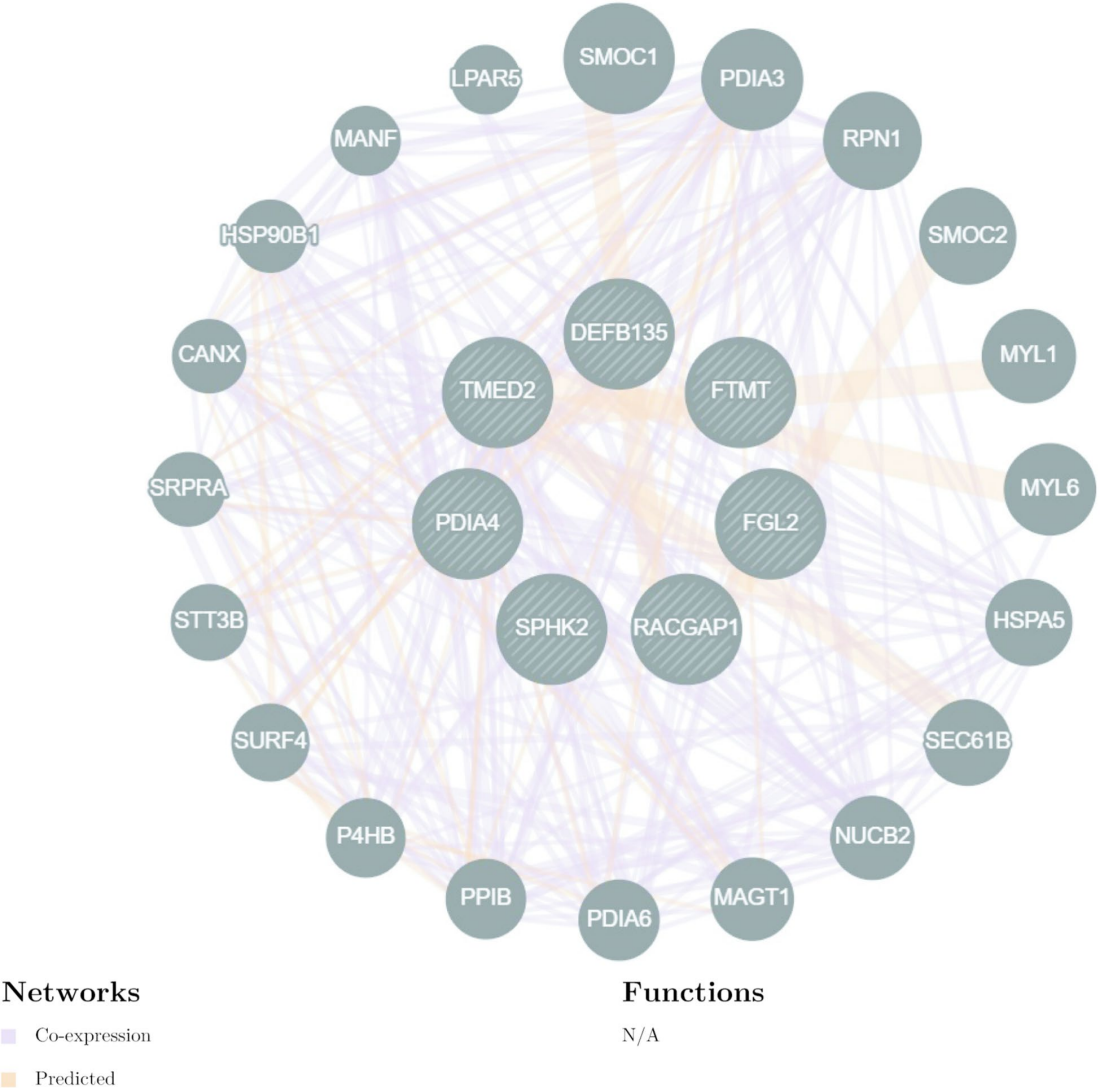
GeneMANIA network analysis of the seven plasma protein-coding genes. The network illustrates functional relationships among these genes, predominantly characterized by co-expression (92.52%) and predicted interactions (7.48%)

KEGG pathway enrichment analysis revealed that the disease-associated plasma proteins were predominantly involved in ferroptosis, porphyrin metabolism, Vibrio cholerae infection, sphingolipid metabolism, VEGF signaling pathway, thyroid hormone synthesis, Fc gamma R-mediated phagocytosis, Sphingolipid signaling pathway, and Apelin signaling pathway. Among these pathways, the highest statistical confidence (*P* < 0.01) was observed for ferroptosis (Figs. 7 and 8). Furthermore, GeneMANIA network analysis demonstrated extensive functional interactions among the plasma protein phenotype genes, with co-expression accounting for 92.52% and predicted interactions representing 7.48% of the gene interaction network (Fig. 9). These findings highlight the complex functional relationships and potential biological mechanisms underlying the roles of these plasma proteins in cervical cancer.

## Discussion

Leveraging large-scale pQTL data and publicly available GWAS summary statistics, we employed MR analysis to investigate the causal relationship between 4,907 plasma proteins and cervical cancer. Our analysis identified seven plasma proteins with significant causal associations: DEFB135, FGL2, FTMT, PDIA4, SPHK2, and TMED2, which may have protective effects against cervical cancer, whereas RACGAP1 may promote cervical carcinogenesis. We conducted sensitivity analyses to minimize the impact of heterogeneity and horizontal pleiotropy on our findings. Furthermore, reverse MR analysis showed no evidence that cervical carcinogenesis drives these plasma proteins' aberrant expression, reinforcing the robustness of the causal relationships. A sensitivity analysis of the inverse MR model revealed that all *P*-values from Cochran's Q test were below 0.05, indicating the presence of heterogeneity. This heterogeneity may result from factors such as horizontal pleiotropy of the instrumental variables, differences in population characteristics between the exposure and outcome cohorts, or measurement errors. In such cases, the random-effects IVW model is typically employed for estimation, as it more effectively accounts for variability in effect sizes. Additionally, methods such as MR-Egger regression and MR-PRESSO should be applied to assess horizontal pleiotropy and identify outliers, thereby ensuring the robustness of causal inference. Functional enrichment analyses using GO and the KEGG revealed that these plasma proteins primarily involve regulation of oxidoreductase activity and ferroptosis pathways. Ferroptosis is characterized by iron-dependent lipid peroxidation, which disrupts cellular membranes and culminates in cell death. This process has been implicated in the pathogenesis of various diseases, including cancer. GeneMANIA analysis indicated that the seven plasma proteins identified as phenotypic genes were situated at the center of the network, forming a dense web of interactions with surrounding genes. These findings provide mechanistic insights into the molecular underpinnings of cervical cancer and highlight potential therapeutic targets, offering a foundation for further experimental and clinical investigations.

The DEFB135 gene encodes defensin beta 135, an antimicrobial peptide critical in modulating immune responses and inflammation, predominantly expressed in human epithelial tissues [25]. Previous studies have shown that DEFB135 expression levels are associated with susceptibility to certain infectious diseases. Cervical cancer is primarily driven by persistent infection with high-risk HPV; it is possible that HPV-mediated downregulation of DEFB135 expression could impair the host's immune defense mechanisms. Although no direct link between DEFB135 and oncogenesis has been established, our MR analysis revealed a significant inverse association between DEFB135 and cervical cancer risk. This finding suggests that DEFB135 may be protective in mitigating disease progression and could represent a potential therapeutic target. However, the precise molecular mechanisms underlying this relationship remain to be elucidated and warrant further investigation.

Fibrinogen-like protein 2 (FGL2), a member of the fibrinogen superfamily, is characterized by its thromboplastin activity and diverse immunomodulatory functions. FGL2 is frequently overexpressed in various tumor tissues [26–28] and has been implicated in the malignant progression of low-grade gliomas (LGGs) to high-grade gliomas (HGGs) [29]. Studies have also demonstrated that FGL2 promotes macrophage polarization and enhances the proliferation of regulatory T cells (Tregs) within the tumor microenvironment, thereby amplifying immunosuppressive activity [30]. In hepatocellular carcinoma, FGL2 overexpression has been closely linked to tumor growth and angiogenesis, suggesting a potential parallel role in cervical cancer [31]. However, the functional significance of FGL2 in cervical cancer remains poorly understood. Our findings indicate that elevated FGL2 expression may have a protective effect against cervical cancer, highlighting the need for further ex vivo and in vivo studies to elucidate the mechanism of this association.

Mitochondrial ferritin (FTMT) is a protein preferentially expressed in cells with high metabolic activity and oxygen-deprived environments, where it functions to sequester iron and mitigate oxidative damage. Fan et al. demonstrated that FTMT expression is inversely correlated with gastric cancer progression [32]. Wu et al. further revealed that FTMT expression is upregulated by hypoxia-inducible factor 1α (HIF-1α), enhancing cellular protection under hypoxic conditions [33]. Additionally, FTMT overexpression can modulate mitochondrial iron availability, potentially contributing to ineffective erythropoiesis, a process implicated in cancer progression [34]. Given that cervical cancer development is strongly associated with HPV infection, which elevates intracellular oxidative stress, FTMT may play a protective role by reducing reactive oxygen species (ROS)-induced DNA damage and suppressing carcinogenesis. These insights align with our findings, suggesting that FTMT may protect against cervical cancer.

Protein disulfide isomerase A4 (PDIA4), an endoplasmic reticulum-resident protein, is critical in protein folding and disulfide bond formation. Previous studies have reported that PDIA4 is aberrantly overexpressed in cervical cancer tissues [35]. Kaplan–Meier survival analyses indicate that elevated PDIA4 expression is associated with poorer clinical outcomes in cervical cancer patients. Functional studies have demonstrated that PDIA4 knockdown significantly suppresses the proliferation and migration of cervical cancer cells. Additionally, evidence suggests that PDIA4 may promote tumor progression by modulating apoptosis and DNA repair pathways, with its overexpression correlating with reduced survival in cervical cancer patients [36]. Nonetheless, an alternative study has demonstrated an association between PDIA4 expression and a reduced risk of certain cancers. Specifically, within the PAX8-AS1 expression quantitative trait locus, particular single-nucleotide polymorphisms are linked to a decreased risk of cervical cancer [37]. This observation aligns with our findings, which suggest that elevated PDIA4 expression may correlate with a lower risk of cervical cancer. The apparent discrepancy may be explained by the fact that somatic overexpression of PDIA4 in tumors likely results from carcinogenic processes, whereas germline-predicted plasma PDIA4 levels reflect an inherent, protective systemic regulatory state. Consequently, while PDIA4 overexpression in cancer is closely associated with tumorigenesis and progression, its expression under normal physiological conditions may confer protective effects. This dual role implies that PDIA4 serves not only as a potential therapeutic target for cancer but also as a protective factor under certain physiological conditions. Understanding the functional mechanisms of PDIA4 across various physiological and pathological contexts will advance the development of more effective cancer treatment strategies and provide novel insights into its role in systemic regulation.

Sphingosine kinase 2 (SPHK2), one of the two isoforms of sphingosine kinase, plays a complex role in cancer biology. Previous studies have demonstrated that SPHK2 regulates intracellular sphingosine-1-phosphate (S1P) levels, influencing cellular growth, survival, and migration [38]. In cervical cancer, SPHK2 overexpression has been associated with enhanced tumor invasiveness and resistance to therapy. At the same time, its inhibition has been shown to suppress the proliferation and migration of cervical cancer cells [39]. Additionally, the pharmacological inhibition of SPHK2 has been reported to sensitize cervical cancer cells to chemotherapeutic agents, potentially overcoming treatment resistance. However, our findings suggest a protective role for SPHK2 in cervical cancer. Early studies suggested that SPHK2 might possess pro-apoptotic and anti-cancer properties, potentially by inducing apoptosis through its BH3 domain. This hypothesis is supported by findings that SPHK2 overexpression inhibits cell growth, induces cell cycle arrest, and exerts anti-proliferative effects. Additionally, SPHK2 may play a protective role in the initiation and progression of cervical cancer by modulating immune surveillance, influencing signaling pathways, or through genetic mechanisms. Therefore, further investigation into the mechanisms underlying the relationship between SPHK2 and cervical cancer is warranted.

Transmembrane p24 transport protein 2 (TMED2) is a key regulator of vesicular protein transport within the cytoplasm. Fang et al. demonstrated that TMED2 is significantly overexpressed in breast cancer tissues at both the mRNA and protein levels [40]. Similarly, elevated TMED2 expression has been observed in head and neck squamous cell carcinoma, a biomarker for poor prognosis [41, 42]. The oncogenic role of TMED2 is further corroborated by studies in other malignancies, including hepatocellular carcinoma, ovarian cancer, lung cancer, and chordoma [43–48]. In cervical cancer, the expression of TMED2 is associated with various clinical and pathological characteristics, including tumor size, lymph node metastasis, and FIGO staging [49]. These correlations suggest that TMED2 may play a significant role in the progression of cervical cancer. However, the relationship between TMED2 and cervical cancer remains poorly characterized. Our findings suggest that TMED2 may function as a protective factor in cervical cancer, highlighting its potential as a novel therapeutic target for this disease.

Rac GTPase-activating protein 1 (RACGAP1) is a specific RhoGAP that regulates Rac1 and Cdc42 signaling, thereby driving tumor progression [50, 51]. Zhang et al. demonstrated that RACGAP1 modulates c-Jun expression via miR-192, and c-Jun, through p-JNK phosphorylation, activates AP-1, promoting the proliferation, migration, and invasion of cervical cancer cells [52]. Additionally, Ruan et al. showed that RACGAP1 speeds up cell cycle progression by regulating CDC25C in cervical cancer cells [53]. Recent studies further revealed that FOXM1 synergizes with RACGAP1, inhibiting apoptosis mediated through the PI3K/AKT signaling pathway [54]. These findings are consistent with our results, suggesting that RACGAP1 may act as a risk factor for cervical cancer and may represent a potential therapeutic target.

Our study has several strengths. First, we leveraged pQTL data from the most extensive proteomics GWAS database, enabling a comprehensive and robust analysis. Second, the MR design effectively minimized confounding factors and reduced the risk of reverse causality, strengthening the validity of our causal inferences. Third, we applied FDR correction and prioritized plasma proteins with consistent odds ratios (OR) directions across analyses, further enhancing the reliability of our findings.

Nevertheless, our study has certain limitations. Firstly, in MR analyses, small sample sizes can undermine the robustness and reproducibility of findings in several ways. (1) Reduced statistical power and diminished estimation precision: Genetic instrumental variables typically explain only a small fraction of the variance in exposure, often less than 1%. With insufficient sample sizes, both the associations between genetic variation and exposure and between genetic variation and outcome exhibit large standard errors, leading to imprecise estimates of causal effects. (2) Increased sensitivity to the validity assumptions of instrumental variables: Many MR methods, such as MR-Egger and the weighted median approach, rely on the assumption of a "valid instrumental variable." When sample sizes are small, it becomes difficult to reliably identify and exclude invalid or partially invalid instrumental variables, which can result in biased causal effect estimates. (3) Reduced reliability of sensitivity analyses: Common MR sensitivity tests-such as heterogeneity tests, MR-Egger intercept tests, and MR-PRESSO-exhibit insufficient statistical power in small sample sizes. This limitation impairs the effective detection of horizontal pleiotropy and other sources of bias. (4) Limited reproducibility of results: Studies with small sample sizes yield wide confidence intervals and highly variable point estimates, making causal estimates prone to inconsistency across different populations or repeated samples. This variability undermines the reproducibility of research findings. (5) Decreased robustness to weak instrumental variables: In small sample contexts, the explanatory power of instrumental variables for the exposure is often weak, as indicated by F-statistics frequently below 10. This weakness amplifies bias from weak instruments and causes causal effect estimates to be biased toward associations observed in conventional observational studies. Secondly, our analysis is limited to European populations and lacks representation from other ethnic groups. This limitation may introduce bias into the estimates and restrict the generalizability of our findings. Therefore, further validation is necessary before extending these results to other racial groups. Third, enrichment analysis serves as a predictive tool, and the accuracy of its results requires further investigation. It is essential to perform additional functional validation, including tissue-specific expression studies and in vivo experiments, to confirm the roles of these proteins as biomarkers or therapeutic targets.

## Conclusion

Our results indicate that DEFB135, FGL2, FTMT, PDIA4, SPHK2, and TMED2 may protect against cervical cancer, whereas RACGAP1 may promote disease pathogenesis. These findings provide a robust theoretical base for identifying potential biomarkers and therapeutic targets for cervical cancer, offering new avenues for further mechanistic and clinical investigations.

## Supplementary Information

Supplementary Material 1.

## Data Availability

No datasets were generated or analysed during the current study.

## Abbreviations

MR: Mendelian randomization
GWAS: Genome-wide association study
IVW: Inverse variance weighting
WM: Weighted median
IVs: Instrumental variables
OR: Odds ratio
FDR: False discovery rate
KEGG: Kyoto Encyclopedia of Genes and Genomes
GO: Gene Ontology
BP: Biological processes
MF: Molecular functions
CC: Cellular components
PPI: Protein–protein interaction
IARC: International Agency for Research on Cancer
HPV: Human papillomavirus
WHO: World Health Organization
SCCA: Squamous cell carcinoma antigen
pQTL: Protein quantitative trait loci
SNPs: Single-nucleotide polymorphisms
LD: Linkage disequilibrium
CC: Cervical cancer
FGL2: Fibrinogen-like protein 2
LGGs: Low-grade gliomas
HGGs: High-grade gliomas
Tregs: Regulatory T cells
FTMT: Mitochondrial ferritin
HIF-1α: Hypoxia-inducible factor 1α
ROS: Reactive oxygen species
PDIA4: Protein disulfide isomerase A4
SPHK2: Sphingosine kinase 2
S1P: Sphingosine-1-phosphate
TMED2: Transmembrane p24 transport protein 2
RACGAP1: Rac GTPase-activating protein 1
